# Outcome of outpatient parenteral antibiotic therapy (OPAT) program at a tertiary care hospital in India

**DOI:** 10.1017/ash.2025.10118

**Published:** 2025-09-05

**Authors:** Anna Rose Varghese, Rajalakshmi Ananthanarayanan, Vettakkara Kandy Muhammed Niyas, Wilson Aloysius Parisavila, Beena Satheesh Vishnu

**Affiliations:** 1 Internal medicine, KIMSHEALTH, Trivandrum, India; 2 Infectious Diseases, KIMSHEALTH, Trivandrum, India

Dear Editor,

We read with interest the article by Deng *et al.*, which describes structured, nurse-driven outpatient parenteral antimicrobial therapy (OPAT) program within an academic healthcare system, and concluded that such an intervention reduced the odds of unplanned OPAT readmissions and reduced costs.^1^ OPAT programs are widely used in developed countries with growing report on high patient satisfaction, clinical- and cost-benefit.^2,3^ Though OPAT is being increasingly used in India, there is not enough data on the outcome. We undertook a pilot study to understand the outcome and safety of OPAT program in Indian settings; also including patient profile and cost benefit. This study was undertaken in a 700 bedded tertiary care hospital in Kerala, south India, from July 2022 to July 2023. Patients who needed OPAT for at least 5 days and fulfilled the following criteria were included: (1) hemodynamically stable and improving (2) had a management plan written by the infectious diseases (ID ) physician regarding IV antibiotics and its duration, based on either susceptibility or syndromic diagnosis, (3) patient with adequate cognitive function to understand and complete OPAT or has a responsible caregiver to facilitate completion of OPAT from an identified OPAT center. Patients were followed up by the OPAT team, led by physicians, a nursing team to monitor venous access related complications, and ID pharmacists to monitor drug related complications. Patient was reviewed by attending clinician or ID physician at the end of therapy. Follow-up was done at 2 weeks post therapy or earlier as indicated. Proportion of patients receiving OPAT getting readmitted within 2 weeks of therapy and outcome at 28 days were looked at. Details of patient demography, adverse events, and cost benefit were studied. OPAT days per patient is considered as days of hospital stay saved. Cost saving was calculated by calculating the cost incurred when treated as OPAT and comparing those to the costs of treating them as inpatients while receiving the same care. During the study period 163 patients received OPAT either in the OPAT ward at our hospital, local hospital, or at home administered by a trained nurse. The details are summarized in Table 1. The median age of the cohort was 62 years (Interquartile Range, IQR: 49–72), comprising 103 males and 60 females. The prevalent comorbidities were as follows: diabetes mellitus (45.4%, *n* = 72), hypertension (35.6%, *n* = 58), malignancy (11%, *n* = 18), chronic liver disease (CLD) (8.6%, *n* = 14), coronary artery disease (8%, *n* = 13), and chronic kidney disease (6.7%, *n* = 11). Primary indications for antibiotic therapy included urinary tract infections (28.2%, *n* = 46), skin and soft tissue infections (22.7%, *n* = 37), bacteremia with no identified source (14.7%, *n* = 24), intraabdominal infections (13.5%, *n* = 22), pneumonia/empyema (9.8%, *n* = 16), bone and joint infections (5.5%, *n* = 9), central nervous system infections (3.7%, *n* = 6), and infective endocarditis (1.8%, *n* = 3). Primary bacteremia without other focus included salmonellosis, leptospirosis. One fourth received antibiotics based on susceptibility, while the rest received based on syndromic diagnosis as decided by ID physician.

**Table 1.** Descriptive characteristics of patients who received OPAT

Total number of patients	163
Age (median, IQR), in years	62 (49–72)
Male: Female	103: 60
***Comorbidities, n (%)***	
Diabetes mellitus	72 (45.4)
Hypertension	58 (35.6)
Malignancy	18 (11)
Chronic liver disease	14 (8.6)
Coronary artery disease	13 (8)
Chronic kidney disease	11 (6.7)
***Indication for OPAT, n (%)***	
Urinary tract infections	46 (28.2)
Skin and soft tissue infections	37 (22.7)
Intraabdominal infections	22 (13.5)
Pneumonia/empyema	16 (9.8)
Bone and joint infections	9 (5.5)
Central nervous system infections	6 (3.7)
Infective endocarditis	3 (1.8)
Bacteremia with no identified source	24 (14.7)
***Microbiological etiology of the infection, n (%)***	
Gram-negative organism	49(30.06)
Gram-positive organism	16(9.8%)
No organism identified	96(58.89)
Mixed growth	2(1.22%)
***Venous access, n (%)***	
Peripheral IV cannula	151 (92.6)
PICC	12 (7.4)
***Site of administration, n (%)***	
OPAT ward/local hospital	109 (66.9)
Home	54 (33.1)
***Antibiotic used, n (%)***	
Cefoperazone-sulbactam	51 (31.3)
Ceftriaxone	47 (28.8)
Ertapenem	35 (21.5)
Piperacillin tazobactam	9 (5.5)
Meropenem	8 (4.9)
Tigecycline	5 (3)
Flucloxacillin	4 (2.45)
Teicoplanin	3 (1.84)
Vancomycin	1 (0.61)
***Outcome***	
Completed therapy without complications	157 (96.3)
Readmission	3 (1.8)
Mortality	3 (1.8)

For the administration of OPAT, a peripheral IV cannula was utilized in 151 patients (92.6%), while a peripherally inserted central catheter (PICC) was employed in 12 patients (7.4%). Home administration of antibiotics was chosen by 54 patients (33.1%), whereas 109 patients (66.9%) received their treatment from a local hospital or from our OPAT ward. Majority received antibiotics for gram-negative infections, as once or twice a day administration. Cefoperazone-sulbactam, ceftriaxone, and ertapenem were the most commonly used antibiotics (see Table 1).

Among 163 patients, 157 (96.3%) successfully completed the treatment and were cured. Three patients (1.8%) were readmitted and 3 expired (1.8%). The patients readmitted were not for infection and were not primarily related to OPAT failure. Notably, there were no hospital readmissions due to IV access or antibiotic-related complications. Among those who expired, one death was attributed to acute coronary syndrome; the other two were due to intraabdominal infection and necrotizing fasciitis, post source control. Both had underlying decompensated CLD and cause of death was considered as ongoing infection or progression of liver disease. The median duration of OPAT was 6.9 days (IQR 5–8 d), and the estimated cost savings per patient was INR 56,000 ($ 674).

We observed that OPAT is a safe option for continuation of IV antibiotics. We noted genito-urinary infection as the most common indication and antibiotic for gram-negative infection were most commonly used. Vascular access related complications, antibiotic adverse events, readmissions related to infection were not noted. Death possibly due to infection occurred in 2 patients who had underlying CLD. OPAT in this group need to be carefully chosen and require close follow-up. In a previous study, referral to sub-acute rehabilitation care and loss of follow-up with ID physician were considered as risk factors for complications related to OPAT.^4^ Identifying risk factors may help in careful selection and planning of OPAT. Moreover, a structured ID-physician and nurse led OPAT program have shown good outcome in previous studies.^5^ In our study, OPAT resulted in cost saving. Indirect cost–benefits through improved efficiency of inpatient bed use by early discharge of patients and reduced rates of healthcare-associated infection need to be addressed and is scope for future study.

Future research on OPAT should focus on understanding implementation barriers in resource limited settings and methods to overcome them, as it’s a low hanging fruit in a stewardship program with many benefits.
